# Effect of negative pressure wound therapy on surgical wound outcomes in colorectal cancer surgery patients: a systematic review and meta-analysis

**DOI:** 10.1186/s12893-026-03652-2

**Published:** 2026-04-11

**Authors:** Mohamed Sherif Ali Ahmed, Mostafa Ismail Mahmoud Hassan, Ahmed Ibrahim, Ahmed Lamey, Mahmoud M. Elsayed, Ahmed Elshaboury, Mohamed Hamouda Elkasaby, Ahmed Abdelrafee

**Affiliations:** 1 Mansoura University Hospital, Al Mansurah, Egypt; 2 Department of General Surgery, Faculty of Medicine, Kafr Elsheikh University, Kafr el-Sheikh, Egypt; 3 Neurosurgery Department, Medical University of South Carolina, Charleston, USA; 4 Faculty of Medicine, Al-Azhar University, Cairo, Egypt; 5 Gastrointestinal Surgery Center, Faculty of Medicine, Mansoura University, Mansoura, Egypt

**Keywords:** Negative Pressure Wound Therapy, NPWT, Colorectal Cancer Surgery, Surgical Site Infection, and Wound Complications

## Abstract

**Background:**

Surgical site infections (SSI) and wound complications are major concerns in colorectal cancer (CRC) surgery. Negative pressure wound therapy (NPWT) has been proposed to reduce these complications, but its efficacy specifically in CRC surgery remains uncertain. This meta-analysis evaluates the impact of NPWT on surgical wound outcomes compared to standard wound care in patients undergoing CRC surgery.

**Methods:**

A systematic search of PubMed, Scopus, Web of Science, and Cochrane Library was conducted up to February 10, 2025, for studies comparing NPWT with standard wound care in CRC surgery patients. The primary outcome was total wound complications. Risk ratios (RR) and mean differences (MD) were pooled using a random-effects model, with heterogeneity assessed using I² statistics.

**Results:**

Six studies involving 343 patients (NPWT: 171, control: 172) were included. NPWT did not significantly reduce total wound complications (RR: 0.67, 95% CI: 0.35–1.26, *P* = 0.21, I² = 66%). Similarly, no significant differences were found for SSI (RR: 0.80, 95% CI: 0.45–1.43, *P* = 0.45), even after sensitivity analysis resolved heterogeneity (RR: 0.62, 95% CI: 0.33–1.17, *P* = 0.14, I² = 0%). NPWT also showed no significant benefit in preventing wound dehiscence (RR: 2.44, *P* = 0.35), seroma (RR: 0.87, *P* = 0.92), or reducing reintervention rates (RR: 0.69, *P* = 0.36). The length of hospital stay was comparable between groups (MD: -0.25 days, *P* = 0.80).

**Conclusion:**

This meta-analysis suggests that NPWT does not significantly reduce wound complications, SSI, or LOS in CRC surgery.

**Supplementary Information:**

The online version contains supplementary material available at 10.1186/s12893-026-03652-2.

## Introduction

 Colorectal cancer (CRC) is a major global health problem. In 2022, cancers of the colorectum ranked among the most commonly diagnosed malignancies worldwide and were a leading cause of cancer death, highlighting the sustained need for effective surgical and perioperative care strategies in this population [1].

Surgical resection remains central to curative CRC management, yet postoperative wound complications are frequent [2]. Colorectal procedures are typically performed in a clean contaminated field, and reported surgical site infection (SSI) rates vary widely across settings, with some series describing rates up to approximately 45% [3, 4]. SSIs are clinically important because they increase resource use and can compromise downstream cancer care [5]. Postoperative infectious complications, including organ and space SSI, have been associated with delayed initiation of adjuvant chemotherapy and worse oncologic prognosis in higher-risk CRC cohorts [6]. Because delays in adjuvant chemotherapy are themselves linked to inferior survival in colon cancer, preventing avoidable postoperative complications remains a priority [7]. SSIs also contribute to longer recovery and may increase the risk of later incisional hernia formation after colorectal surgery [8, 9].

The pathogenesis of SSI is multifactorial, reflecting patient factors, operative characteristics, and perioperative processes of care [4]. Multiple evidence-based prevention measures are therefore routinely implemented, including appropriate antimicrobial prophylaxis, meticulous aseptic technique, and broader bundle-based approaches to reduce modifiable risks [10, 11]. Despite these strategies, SSI remains common after colorectal operations, which has driven interest in adjunctive interventions that can further optimize incision management, particularly for patients and procedures at increased risk [4, 11].

Negative pressure wound therapy (NPWT) has emerged as a potential adjunct to improve wound healing and reduce infection risk [12]. NPWT applies controlled sub-atmospheric pressure through a sealed dressing connected to a suction source, promoting fluid removal and modifying the wound environment [13]. Experimental and clinical work suggests that NPWT can support healing through mechanisms that include exudate control, reduction of tissue oedema, and improved local perfusion [14, 15]. In closed surgical incisions, incisional NPWT has been evaluated as a prophylactic strategy to reduce SSI and related complications when compared with standard dressings. Evidence syntheses across surgical specialties suggest that NPWT probably reduces SSI after primary closure, although the magnitude of benefit may vary by procedure type and baseline risk [13, 16].

International guidance reflects this uncertainty. World Health Organization (WHO) guidance includes a conditional recommendation that prophylactic NPWT may be used on primarily closed incisions in high-risk wounds while emphasizing limitations in the certainty of evidence [17]. More recent trials have also challenged assumptions of universal benefit. For example, the SUNRRISE randomized clinical trial reported no reduction in SSI with incisional NPWT compared with standard dressings in adults undergoing emergency laparotomy [18]. In CRC surgery, where contamination risk and perineal or abdominal wound morbidity can be substantial, the effectiveness of NPWT remains an open and clinically important question, and available evidence has been heterogeneous across populations and operative contexts [3, 4, 19].

Accordingly, this systematic review and meta-analysis aimed to evaluate the effect of NPWT on postoperative surgical wound outcomes in patients undergoing CRC surgery, compared with standard wound dressings, to inform evidence-based incision management in this high-risk setting.

## Methods

This study followed the methodologies outlined in the Cochrane Handbook of Systematic Reviews on Interventions [20]. The study protocol was registered prospectively in the PROSPERO (International Prospective Register of Systematic Reviews) (Registration ID: CRD420251166203). The publication was prepared in accordance with the guidelines set forth by the Preferred Reporting Items for Systematic Reviews and Meta-Analyses (PRISMA) statement [21]. The work was reported in accordance with AMSTAR-2 (A Measurement Tool to Assess Systematic Reviews 2) guidelines for assessing the methodological quality of systematic reviews [22].

### Requirements for eligibility

This meta-analysis included studies that directly compared NPWT with standard wound care in the context of surgical wound management for patients undergoing colorectal cancer surgery. The main outcomes evaluated included total wound complications, surgical site infection (SSI), wound dehiscence, seroma, rates of reintervention, and duration of hospital stay. Eligible study designs comprised randomized controlled trials (RCTs), cohort studies, and case-control studies. To ensure clinical relevance and precision in our analysis, we specifically considered key surgical characteristics when defining our study population. Our focus included patients undergoing both open and minimally invasive colorectal cancer procedures in elective settings. We included studies evaluating NPWT application to both abdominal incisions and perineal wounds following abdominoperineal resection (APR). Given the particular relevance to perineal wound healing, we also documented the use of neoadjuvant therapy across included studies, as this represents a significant risk factor for wound complications in APR patients. Studies were excluded if they lacked sufficient outcome data, were limited to conference abstracts, case reports, case series, or non-human research, or were published in languages other than English.

### Literature searching and study selection

We conducted a comprehensive search across four major electronic databases: PubMed, Scopus, Web of Science, and the Cochrane Library, covering all studies published until February 8, 2025. The search strategy included terms related to NPWT and colorectal cancer. The complete search strategy for each database is provided in *Supplementary Table 1*. All retrieved records were imported into EndNote for organization and deduplication. Two independent reviewers conducted a two-step screening process. Initially, titles and abstracts were screened to exclude irrelevant studies. The remaining full-text articles were assessed for eligibility based on predefined inclusion and exclusion criteria. Reference lists of included studies and relevant reviews were also screened manually to identify additional eligible studies. Furthermore, to minimize selection bias, a dual-review process was employed at each stage of study selection and data extraction, with discrepancies resolved through consensus or third-party adjudication.

### Quality assessment

The quality of the cohort studies was assessed according to the Newcastle-Ottawa quality assessment scale [23], case-control studies were assessed according to NOS for case-control studies [23], and RCTs were assessed using the Cochrane Risk of Bias 2 (ROB 2) tool [24]. Two authors independently evaluated the quality of the included studies, and in case of any disagreement, the first author made the final decision.

### Data extraction and outcomes

Two reviewers independently extracted data using a predefined form, collecting information on study characteristics (authors, year, design, and country), patient characteristics (sample size, mean age, BMI, and comorbidities), intervention details (NPWT versus standard wound care), and clinical outcomes, including SSI, wound dehiscence, seroma, reintervention rates, total wound complications, and LOS.

For the outcome “total wound complications,” we defined this as a composite endpoint for the purpose of this review, encompassing all reported surgical wound-related adverse events from the included studies. This composite included the individual outcomes of SSI, wound dehiscence, seroma, and need for reintervention, as these were the most consistently reported wound-specific complications across studies. No additional wound-related outcomes beyond these four components were pooled into the “total wound complications” metric. This approach was chosen to provide a comprehensive assessment of overall wound morbidity.

For the outcome “need for reintervention,” this was defined as a return to the operating theatre specifically for the management of wound-related complications, such as surgical drainage of an abscess, evacuation of a hematoma, or secondary closure of a dehisced wound. This definition did not include other, non-wound-related reasons for reoperation (e.g., anastomotic leak, bowel obstruction) or percutaneous image-guided drainage procedures, to maintain a specific focus on interventions directly addressing the surgical wound.

To ensure a transparent assessment of potential heterogeneity in outcome measurement, we systematically extracted data on how each included study defined and measured the primary wound complication outcomes (SSI, wound dehiscence, seroma, reintervention). These definitions and assessment methods are summarized in *Supplementary Table 2*. As noted in the table, several studies did not report a specific definition for key outcomes like SSI or wound dehiscence, relying instead on clinical diagnosis. The variability in definitions and the lack of standardized reporting for some outcomes are acknowledged as a potential limitation and are discussed further in the context of our findings.

The included studies exhibited variability in their definitions of surgical site infection (SSI). While some studies explicitly used standardized criteria such as those from the Centers for Disease Control and Prevention (CDC), others defined SSI based on clinical signs or did not report a specific definition. To ensure a consistent and objective analysis, we accepted the SSI definition as reported by the original study authors for data extraction and pooling. We prioritized the inclusion of studies where SSI was a clearly reported outcome, irrespective of the specific definition used, to allow for a comprehensive assessment of this key endpoint. Furthermore, a post-hoc sensitivity analysis was performed by excluding studies that did not explicitly reference a standardized SSI definition (e.g., CDC criteria), which did not alter the overall non-significant finding for SSI.

### Statistical analysis

Statistical analyses were conducted using Review Manager (RevMan, version 5.4, The Cochrane Collaboration). For continuous outcomes, mean differences (MD) with 95% confidence intervals (CI) were calculated. For dichotomous outcomes, relative risks (RR) with 95% CI were reported. Heterogeneity across studies was assessed using the Chi-squared (χ²) test and quantified with the I² statistic. Heterogeneity was categorized as low (I² = 0%–25%), moderate (I² = 26%–50%), or high (I² >50%). A random-effects model was applied. Publication bias was not tested, as the number of included studies was less than 10, indicating unreliable publication bias results as reported by Egger et al. [25].

## Results

### Literature search

**Fig. 1:**
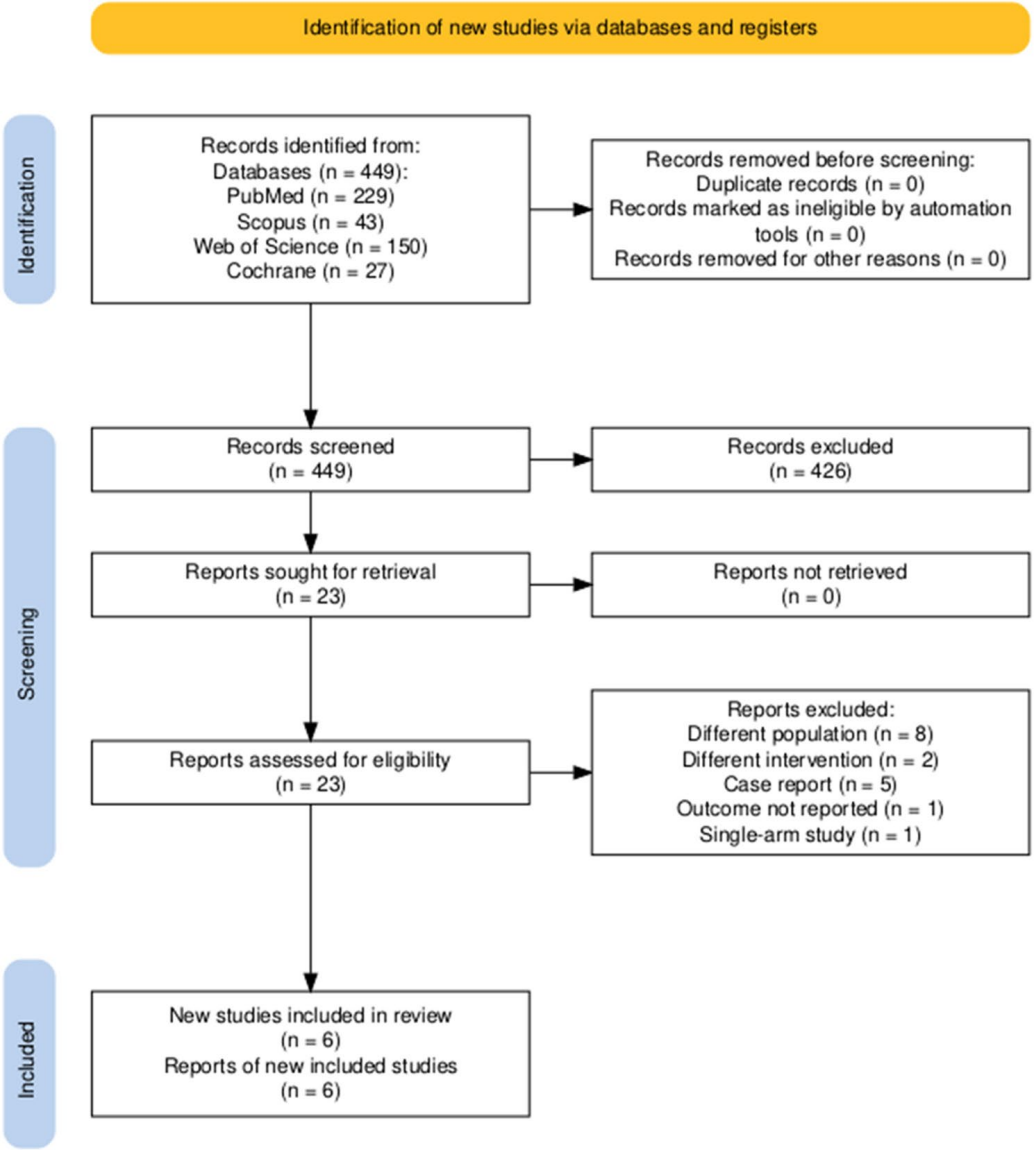
PRISMA flow diagram of the included studies

A systematic search of four electronic databases (PubMed, Scopus, Web of Science, and Cochrane Library) identified 449 records (PubMed: 229, Scopus: 43, Web of Science: 150, Cochrane: 27). No duplicate records were identified or removed before the screening. Following title and abstract screening, 426 records were excluded based on relevance to the research question. The remaining 23 studies underwent full-text assessment for eligibility. Upon full-text evaluation, 17 studies were excluded for the following reasons: different population (*n* = 8), different intervention (*n* = 2), case reports (*n* = 5), outcome not reported (*n* = 1), and single-arm study design (*n* = 1). Consequently, six studies met the inclusion criteria and were included in the final review. The selection process is visualized in the PRISMA flow diagram Fig.1.

### Characteristics of the included studies

Table 1 summarizes the characteristics of the included studies. Six studies were included, encompassing a total of 343 patients, with 171 receiving NPWT and 172 undergoing standard wound dressing. The studies were conducted in the UK, the Netherlands, China, Finland, and Turkey. The specific methodological designs were as follows: two were prospective cohort studies, one was a randomized controlled trial, one was a prospective case series, one was a retrospective cohort study, and one was a retrospective case-control study. The included studies encompassed diverse surgical approaches relevant to colorectal cancer surgery. Specifically, the population consisted of patients undergoing both open and minimally invasive procedures, with all studies conducted in elective settings. Three studies focused specifically on perineal wounds following APR, while others included abdominal incisions. The use of neoadjuvant therapy, particularly relevant for perineal wound healing outcomes, varied across studies from 20% to 57%. Table 2 summarizes the baseline characteristics of the included studies. The median or mean age of participants ranged from 64.5 to 78.7 years, with the proportion of male patients varying between 45% and 90.5%. BMI was reported in some studies, ranging from 23.9 to 26.5 kg/m². Common comorbidities included hypertension, diabetes, hypoalbuminemia, chronic obstructive pulmonary disease, and cardiovascular disease. Neoadjuvant therapy varied from 20% to 57%. Follow-up durations ranged from 30 days to six months.

**Table 1:** Baseline characteristics of the included studies

Study	Group	Number of patients	Age (Mean ± SD)	Sex (Male/Female)	BMI (Mean ± SD)	Comorbidities (N, %)	Neoadjuvant Therapy (N, %)	Tumor Location
Yang 2020​	NPWT	11	73.18±10.67	7/4(63.64%/36.36%)	23.90±3.01	Hypertension: 1 (9.09%), Diabetes: 1 (9.09%), Hypoalbuminemia (<3.5 g/dL): 2 (18.18%), COPD: 2 (8.33%), Congestive Heart Disease: 1 (4.17%)	NA	Rectal
	Control	13	69.85±6.73	8/5(61.54%/38.46%)	24.31±3.82	Hypertension: 2 (15.38%), Diabetes: 2 (15.38%), Hypoalbuminemia (<3.5 g/dL): 3 (23.08%), COPD: 1 (5%), Congestive Heart Disease: 0 (0%)	NA	Rectal
Salmenkylä 2022​	NPWT	21	71	19/2 (90.5%/9.5%)	BMI > 30: 2 (9.5%)	Neoadjuvant radiation: 12 (57%), T3: 11 (52%), T4: 2 (9.5%)	12 (57%)	Rectal
	Control	21	69	13/8 (62%/38%)	BMI > 30: 2 (9.5%)	Neoadjuvant radiation: 12 (57%), T3: 11 (52%), T4: 2 (9.5%)	12 (57%)	Rectal
van der Valk 2017​	NPWT	10	65.4 (51–83)	6/4 (60%/40%)	26.46	Cardiovascular comorbidity: 5 (50%)	CRT: 4 (40%), RT: 3 (30%)	Rectal
	Control	10	66.6 (45–79)	6/4 (60%/40%)	26.05	Cardiovascular comorbidity: 5 (50%)	CRT: 2 (20%), RT: 3 (30%)	Rectal
Liu 2021​	NPWT	76	78.04 ± 5.74	34/42 (45%/55%)	NA	Anaemia: 15 (19.7%), Diabetes: 18 (23.7%), Hypertension: 33 (43.4%)	NA	Right colon, rectum, sigmoid
	Control	74	78.74 ± 6.56	40/34 (54%/46%)	NA	Anemia: 12 (16.2%), Diabetes: 8 (10.8%), Hypertension: 16 (21.6%)	NA	Right colon, rectum, sigmoid
Kaçmaz 2022​	NPWT	24	67.4 ± 9.1	14/10 (58.3%/41.7%)	NA	Diabetes: 13 (54.2%), COPD: 1 (4.2%)	6 (25%)	Colon, Rectal
	Control	26	64.5 ± 9.1	13/13 (50%/50%)	NA	Diabetes: 9 (34.6%), COPD: 2 (7.7%)	6 (25%)	Colon, Rectal
Sumrien 2016	NPWT	32	NA	24/8	NA	Preoperative chemoradiotherapy 13 (41%)	NA	Rectal
	Control	25	NA	19/6	NA	Preoperative chemoradiotherapy 10 (25%)	NA	Rectal
*Abbreviations*: *NPWT* Negative-Pressure Wound Therapy, *BMI* Body Mass Index, *COPD* Chronic Obstructive Pulmonary Disease, *CRT* Chemoradiotherapy, *RT* Radiotherapy, *NA* Not Available								

**Table 2:** Characteristics of the included studies

Study	Sumrien et al. 2016	van der Valk et al. 2017	Yang et al. 2020	Salmenkylä et al.2022	Kaçmaz et al.2022	Liu et al. 2021
Design	Prospective	Prospective	Prospective	Case-control	RCT	Retrospective
Sample Size	57	20	24	42	50	150
Country	UK	Netherlands	China	Finland	Turkey	China
Intervention details	NPWT applied to closed perineal wounds. 125 mmHg for closed perineal wounds	NPWT (-80 mmHg) for 7 days. A portable NPWT device applied for 7 days.	NPWT. NPWT applied after DPWC	NPWT (Avelle®, Convatec™) at 80 mmHg. NPWT with biological mesh and local flap closure.	NPWT applied for 7 days postoperatively	NPWT
Comparator	Standard perineal wound closure with dressings	Standard dressing	Standard dressing	Standard dressing	Standard dressing with Sterile gauze	Standard dressing
Outcome Measures	Wound healing, complications	Wound infection, healing time	Wound healing, infection rate	SSI, wound healing	Wound complications, hospital stay	Wound healing, hospital stay
Details of Procedure	ELAPE with biological mesh closure	Laparoscopic APR for rectal cancer	APR for rectal carcinoma	APR for rectal adenocarcinoma	Open colorectal cancer surgery	Radical surgery for colorectal cancer
Antibiotic Therapy	Yes	Yes	Yes	Yes	Yes	Yes
Bowel Preparation	Not reported	Not reported	Not reported	Yes	Yes	Yes
SSI Definition	Not reported	Not reported	Not reported	Defined by CDC criteria	Defined based on clinical signs	Not reported
Follow-up	30 days	Median follow-up: 13 weeks	3 months	Median follow-up: 6 months	30 days	Postoperative 30-day follow-up
*Abbreviations*: *NPWT *Negative-Pressure Wound Therapy, *APR *Abdominoperineal Resection, *DPWC *Direct Perineal Wound Closure, *CPWC *Conventional Perineal Wound Closure, *SSI *Surgical Site Infection, *CRC *Colorectal Cancer, and *CDC *Centers for Disease Control and Prevention						

### Quality of the included studies

**Fig. 2:**
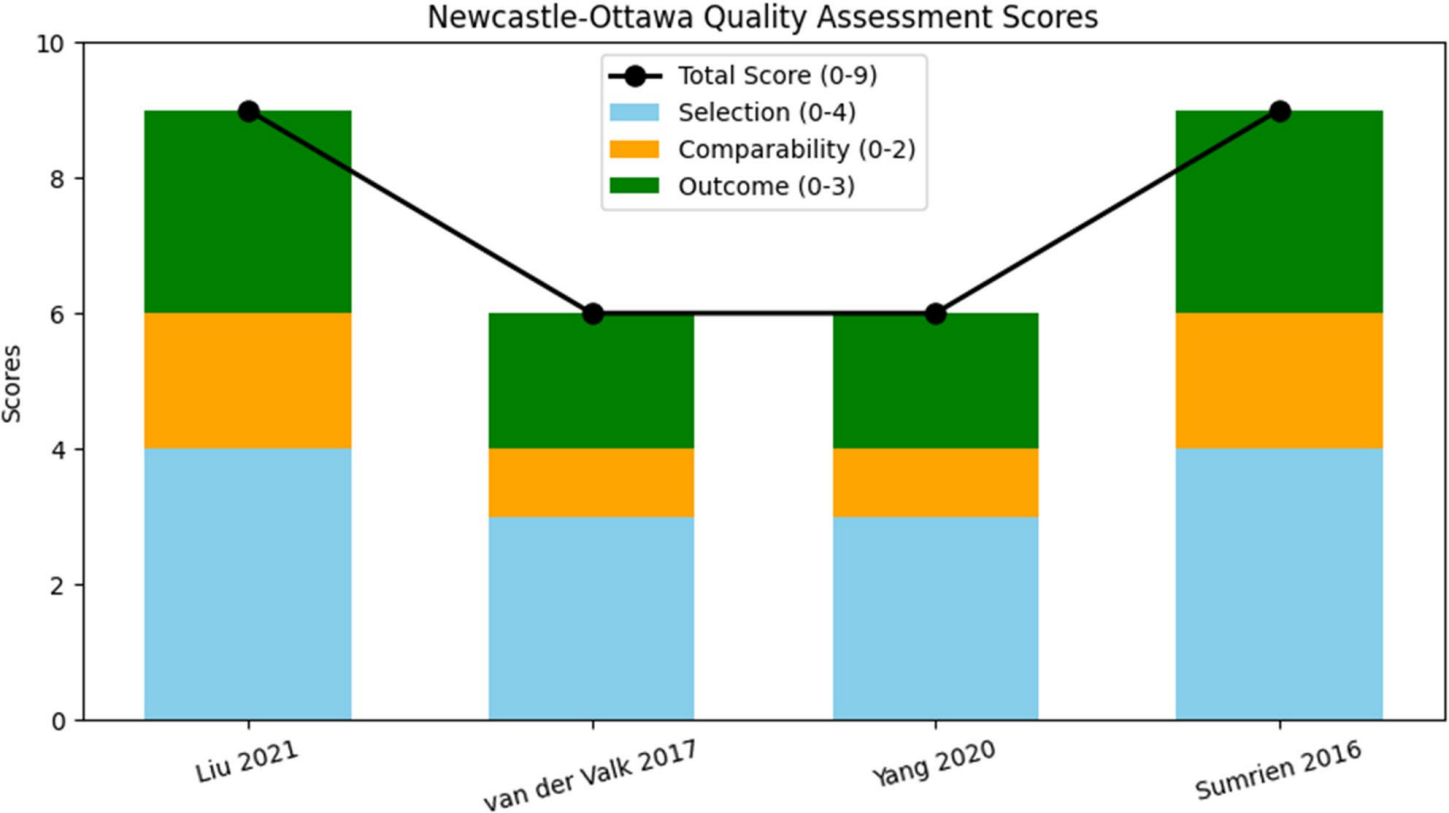
Methodological quality across the cohort studies, according to the Newcastle-Ottawa scale

The NOS assessment indicated that Liu 2021 and Sumrien 2016 [26, 27] had the highest quality scores (9/9), with strong selection methods, well-matched cohorts, and complete outcome reporting. Van der Valk 2017 and Yang 2020 scored 6/9, concluding overall moderate quality, with some concerns due to incomplete confounder adjustments and comparability [28, 29]; Fig. 2.

**Fig. 3:**
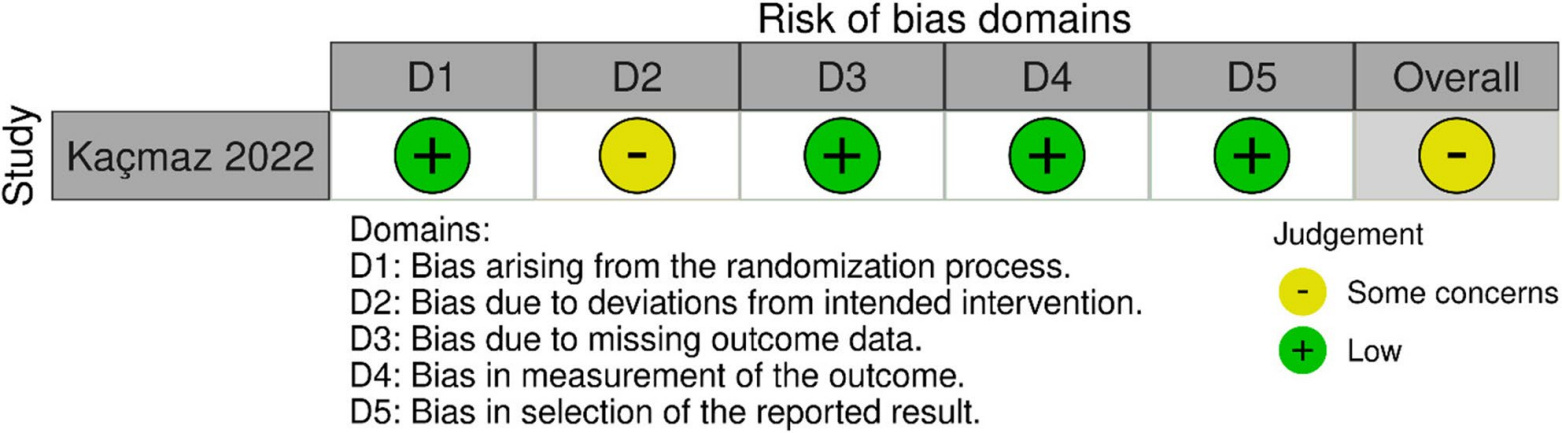
Quality assessment of randomized-controlled trials according to the Cochrane ROB 2 tool

The Kaçmaz 2022 study [30] had an overall risk of bias of some concerns due to the lack of blinding, which may have introduced performance bias. However, the study had a low risk of bias in randomization, missing data, outcome measurement, and reporting, ensuring reliable results; Fig. 3.

**Fig. 4:**
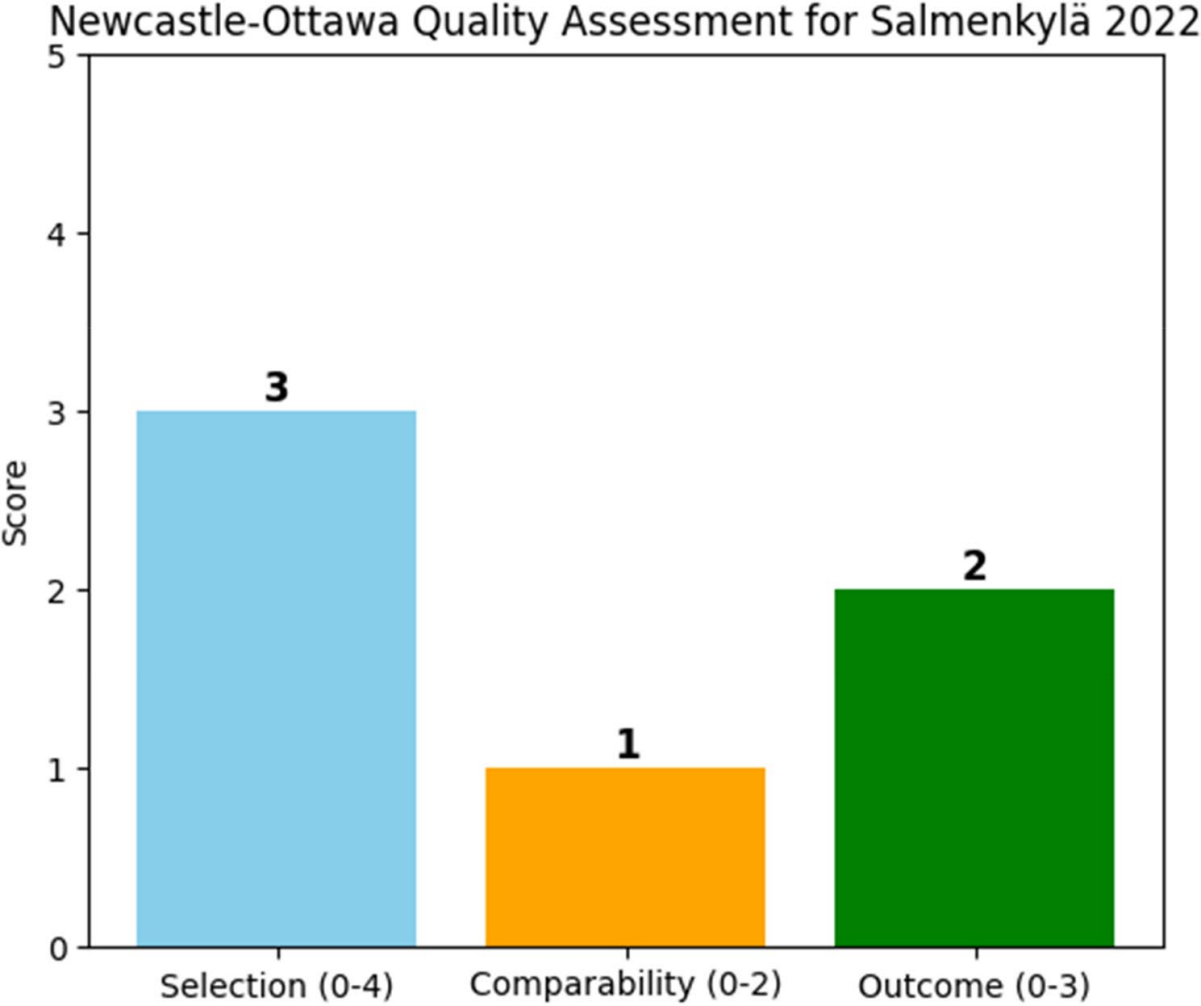
Methodological quality across case-control studies, according to the Newcastle-Ottawa scale

The NOS for case-control studies assessment for Salmenkylä 2022 [31] rated it as moderate quality (6/9); Fig. 4. The study had well-defined cases and controls (3/4) but used retrospective control selection. Matching was adequate (1/2), though some confounders were unadjusted. Outcome assessment was standardized (2/3), but missing data introduced bias.

### Clinical outcomes

#### Total wound complications

**Fig. 5:**
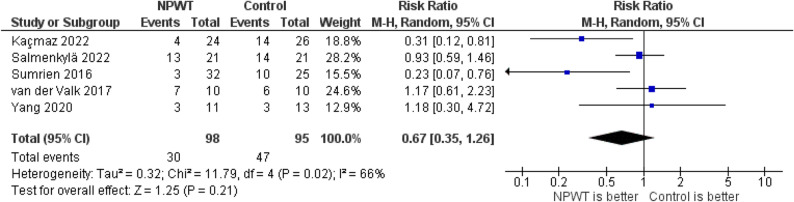
Forest plot of total wound complications

The pooled analysis of five studies evaluating total wound complications demonstrated no statistically significant difference between NPWT and standard wound dressing. The RR was 0.67 (95% CI: 0.35–1.26; *P* = 0.21), with moderate-to-high heterogeneity (I² = 66%), Fig. 5. Leave-one-out analysis did not result in resolved heterogeneity.

#### Surgical site infection

**Fig. 6:**
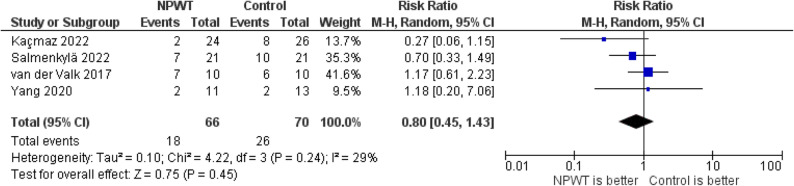
Forest plot of SSI

The pooled analysis of four studies on SSI demonstrated no statistically significant difference between NPWT and standard wound dressing. The RR was 0.80 (95% CI: 0.45–1.43; *P* = 0.45), with low heterogeneity (I² = 29%), Fig. 6.

**Fig. 7:**
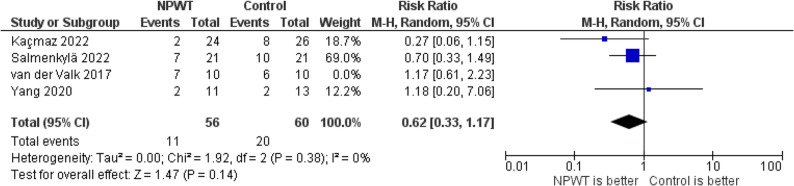
Forest plot of SSI after excluding van der Valk 2017

The pooled analysis of SSI after excluding resolving van der Valk 2017 showed resolved heterogeneity and demonstrated no statistically significant difference between NPWT and standard wound dressing. The RR was 0.62 (95% CI: 0.33–1.17; *P* = 0.14), with no heterogeneity (I² = 0%), Fig. 7.

#### Seroma

**Fig. 8:**
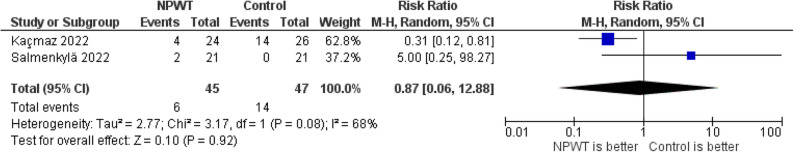
Forest plot of Seroma

The pooled analysis of two studies evaluating seroma formation demonstrated no statistically significant difference between NPWT and standard wound dressing. The RR was 0.87 (95% CI: 0.06–12.88; *P* = 0.92), with high heterogeneity (I² = 68%), Fig. 8.

#### Wound dehiscence

**Fig. 9:**
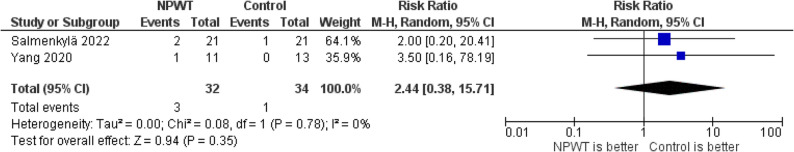
Forest plot of wound dehiscence

The pooled analysis of two studies evaluating wound dehiscence demonstrated no statistically significant difference between NPWT and standard wound dressing. The RR was 2.44 (95% CI: 0.38–15.71; *P* = 0.35), with no heterogeneity (I² = 0%), Fig. 9.

#### Need for reintervention

**Fig. 10:**
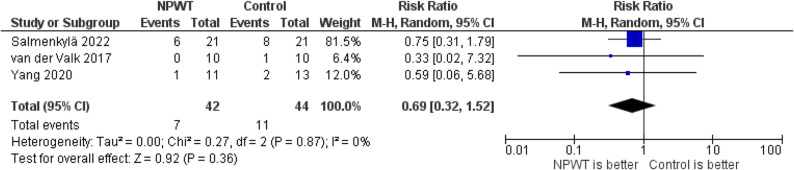
Forest plot of the need for reintervention

The pooled analysis of three studies evaluating the need for reintervention demonstrated no statistically significant difference between NPWT and standard wound dressing. The RR was 0.69 (95% CI: 0.32–1.52; *P* = 0.36), with no heterogeneity (I² = 0%), Fig. 10.

#### Length of hospital stay

**Fig. 11:**
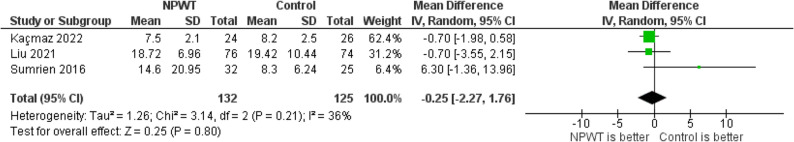
Forest plot of the length of hospital stay

The pooled analysis of three studies evaluating LOS demonstrated no statistically significant difference between NPWT and standard wound dressing. The mean difference was − 0.25 days (95% CI: -2.27 to 1.76; *P* = 0.80), with moderate heterogeneity (I² = 36%), Fig. 11.

**Fig. 12:**
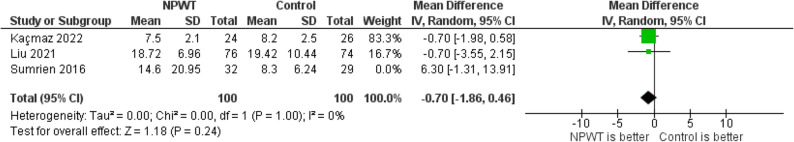
Forest plot of length of hospital stay after excluding Sumrien 2016

To resolve this heterogeneity, a leave-one-out sensitivity analysis was performed. Excluding Sumrien 2016 reduced heterogeneity to I² = 0%, with a recalculated MD of -0.70 days (95% CI: -1.86 to 0.46; *P* = 0.24); Fig. 12.

## Discussion

This systematic review and meta-analysis synthesized six comparative studies, including 343 patients, and evaluated incisional NPWT versus standard wound dressing after elective colorectal cancer surgery. The pooled results showed no statistically significant differences in total wound complications, SSI, seroma, wound dehiscence, need for reintervention, or LOS, and the estimates were generally imprecise, consistent with limited information size and heterogeneous clinical settings.

For total wound complications, the pooled effect did not show a clear benefit, and heterogeneity remained moderate to high, which suggests that differences in incision type, baseline risk, and perioperative protocols likely influenced observed effects. Clinically, this means routine incisional NPWT for all colorectal cancer incisions cannot be supported based on current comparative evidence. A key biological explanation is that abdominal incisions and perineal wounds after abdominoperineal resection (APR) are driven by different mechanisms of failure, so combining them can dilute a benefit that might be confined to one anatomical site [32].

For SSI, our pooled estimate also remained neutral, despite favoring NPWT directionally in some analyses, and sensitivity analyses suggested that individual studies could influence heterogeneity without changing the overall conclusion. Biologically, incisional NPWT can plausibly reduce superficial SSI by sealing the incision, limiting external contamination, reducing edema, and removing exudate that can support bacterial proliferation [33]. However, colorectal surgery carries a meaningful burden of deep incisional and organ space infection pathways, and these may be less responsive to an incision-level intervention when risk is dominated by bowel flora, pelvic dead space, and radiotherapy-related tissue injury [34]. This biological mismatch may help explain why colorectal-specific randomized evidence has been mixed, including the NEPTUNE randomized trial that found no SSI reduction with incisional NPWT compared with standard dressing [35]. When compared with broader literature, our neutral findings differ from several meta-analyses across mixed surgical populations that report fewer SSIs with prophylactic incisional NPWT, with effect estimates commonly indicating a relative reduction [36–38]. A plausible cause of this discrepancy is that many mixed surgery meta-analyses include procedures and populations with higher baseline soft tissue risk profiles, where a barrier and edema-reducing dressing effect may translate more reliably into fewer superficial infections.

In colorectal cancer surgery, the incremental effect of incisional NPWT may be smaller when modern prevention bundles are already applied, which is consistent with NEPTUNE and with the limited signal seen in our pooled estimates [35]. In addition, observational colorectal cohorts are vulnerable to confounding by indication, because NPWT may be preferentially used in higher-risk patients, which can bias estimates toward the null if adjustment is incomplete.

For seroma, the pooled estimate did not show a significant difference and was extremely imprecise, which limits clinical inference. Mechanistically, negative pressure could reduce seroma by collapsing potential space and continuously evacuating fluid, and this has been proposed as a key benefit in closed incision management [39]. In colorectal surgery, however, drains, hemostasis, and closure technique may dominate seroma formation more than dressing choice, and inconsistent definitions and ascertainment can further obscure true effects [40].

For wound dehiscence, the pooled analysis did not show a statistically significant difference, but estimates were imprecise and based on few studies, so a clinically important benefit or harm cannot be excluded. Incisional NPWT may theoretically reduce separation by splinting the incision and distributing mechanical forces, but device-related skin blistering and edge maceration have also been reported and may offset any mechanical advantage in some patients [41]. Systemic drivers of dehiscence, such as malnutrition, diabetes, and steroid exposure, are unlikely to be corrected by an incision dressing alone, which further limits the expected effect size [42].

For the need for reintervention, the pooled estimate remained neutral, which is clinically plausible because many reinterventions after colorectal surgery are driven by pelvic collections, anastomotic complications, or intra-abdominal sepsis rather than superficial incision problems alone [43]. Accordingly, an incision-focused intervention may have limited impact on reintervention unless it can also reduce deep infection pathways, which is less certain in APR settings where pelvic dead space is central [43].

For LOS, no meaningful difference was observed, and this is clinically expected because enhanced recovery pathways shorten admission, while many wound complications present after discharge [44]. LOS is also influenced by bowel function, pain control, and medical complications that are largely independent of incision dressing selection, which further weakens LOS as a sensitive endpoint for incisional NPWT benefit [45, 46]. When focusing on perineal wounds after APR, the existing evidence suggests a possible role for prophylactic incisional NPWT, but certainty remains limited due to small samples, heterogeneous definitions, and predominance of nonrandomized designs [47]. In this context, dressing level approaches should be interpreted alongside other strategies that address pelvic dead space and perineal reconstruction, because these may be more directly linked to deep perineal complications.

Meta-analyses of flap reconstruction often report fewer perineal wound complications compared with primary closure, although tradeoffs include operative time, donor site morbidity, and resource needs [48, 49]. Randomized evidence for other options, such as biological mesh, has focused mainly on structural outcomes like perineal hernia rather than consistent short-term reduction in wound complications, which illustrates that different interventions target different failure mechanisms [50]. Therefore, neutral pooled effects for incisional NPWT in mixed colorectal incisions can still be compatible with benefit in narrowly defined high-risk perineal subgroups, but this remains unproven.

Overall, the most clinically consistent interpretation is that routine incisional NPWT for all elective colorectal cancer surgery incisions has uncertain benefit, with current evidence excluding neither a modest benefit in selected high-risk patients nor a null effect in unselected populations. Selective use based on baseline risk factors such as obesity, diabetes, radiotherapy exposure, and complex perineal closure may be more biologically and clinically rational than universal use, while recognizing that comparative evidence remains limited.

### Clinical implications

Current guidance generally supports selective rather than universal use of prophylactic incisional NPWT on primarily closed incisions [51]. The World Health Organization guideline suggests considering prophylactic incisional NPWT in high-risk patients, but recommendations are conditional and based on low certainty evidence [51]. National Institute for Health and Care Excellence (NICE) medical technologies guidance supports the use of single-use NPWT systems for closed incisions in patients at higher risk of SSI, emphasizing targeted adoption rather than routine application for every patient [52]. In hospital infection prevention guidance, incisional NPWT is framed as an adjunct in selected settings with variable certainty by procedure type. For colorectal surgery specifically, guidelines emphasize measures with stronger and more consistent evidence for reducing SSI, including appropriate systemic antibiotic prophylaxis and bowel preparation strategies that reduce intraluminal microbial burden in selected patients. The enhanced recovery after surgery (ERAS) colorectal guidance does not position incisional NPWT as a core universal element of care, which is concordant with the neutral effect seen in colorectal randomized evidence and with our pooled results [53]. Importantly, the included evidence spans heterogeneous incision contexts (abdominal incisions and perineal APR wounds); therefore, pooled estimates should be interpreted as an average across sites and may dilute site-specific effects in higher-risk wound environments. Practically, our findings suggest that if incisional NPWT is used, it should be considered as a risk-targeted add-on rather than a substitute for established colorectal SSI prevention bundles. Given device costs and the need for appropriate application and monitoring, risk stratification and local cost effectiveness should guide implementation decisions.

### Strengths, limitations, and recommendations

Strengths include a multi-database search strategy, explicit eligibility criteria, and structured quality appraisal across study designs. We evaluated outcomes that are clinically meaningful, including reintervention and LOS, and conducted sensitivity analyses for key endpoints, which support transparent interpretation. Limitations are substantial and likely explain the imprecision of pooled effects. The evidence base is small and largely observational, increasing vulnerability to confounding and selection bias, particularly confounding by indication. Several outcomes rely on two to three studies, producing very wide confidence intervals and limiting the ability to detect modest but clinically relevant effects. Clinical heterogeneity is considerable, including mixed abdominal and perineal incisions, variable radiotherapy exposure, different NPWT systems and durations, and inconsistent outcome definitions and follow-up windows. Finally, SSI subtype reporting was limited in several studies, and pooling across superficial, deep, and organ space events can obscure mechanism-aligned effects. Future research should prioritize adequately powered randomized trials in well-defined colorectal subgroups, with stratification by incision site, especially perineal wounds after APR. Trials should use standardized SSI definitions and report superficial, deep, and organ space infections separately to align outcomes with biological plausibility. Studies should also capture device-related adverse events such as blistering, patient-centered outcomes, and cost effectiveness, because these factors influence real-world adoption.

## Conclusion

In elective colorectal cancer surgery, current comparative evidence does not demonstrate statistically significant reductions in wound complications with incisional NPWT compared with standard dressings. Any benefit is uncertain and likely modest, and may be limited to selected high-risk patients or specific incision types such as perineal wounds after APR. High-quality randomized trials with standardized outcomes and incision site stratification are needed to define the role of incisional NPWT within contemporary colorectal surgery pathways.

## Supplementary Information

Supplementary Material 1.

Supplementary Material 2.

## Data Availability

All data generated or analyzed during this study are included in this published article.

## Abbreviations

CRC: Colorectal cancer
SSI: Surgical site infections
NPWT: Negative pressure wound therapy
WHO: World Health Organization
APR: Abdominoperineal resection
PROSPERO: International prospective register of systematic reviews
PRISMA: Preferred Reporting Items for Systematic Reviews and Meta-Analyses
AMSTAR-2: A Measurement Tool to Assess Systematic Reviews 2
RCTs: Randomized controlled trials
NOS: Newcastle–Ottawa Scale
ROB 2: Cochrane Risk of Bias 2
BMI: Body mass index
LOS: Length of hospital stay
CDC: Centers for Disease Control and Prevention
RevMan: Review Manager
MD: Mean difference
CI: Confidence interval
RR: Risk ratio
χ²: Chi-squared
I²: I-squared statistic
NICE: National Institute for Health and Care Excellence
ERAS: Enhanced Recovery After Surgery
