# Supplementary material for: Effect of negative pressure wound therapy on surgical wound outcomes in colorectal cancer surgery patients: a systematic review and meta-analysis
Source: BMC Surg. 2026 Apr 11;26:278. doi: 10.1186/s12893-026-03652-2 (PMC13088833; doi:10.1186/s12893-026-03652-2)
Supplement: Supplementary file 1 — Supplementary Material 1. [file 12893_2026_3652_MOESM1_ESM.docx]

| Supplementary Table 1. Search strategy for each database | | Number of results |
| --- | --- | --- |
| Database | **Search strategy** |  |
| PubMed | (((((prophylactic negative-pressure wound therapy) OR (pNPWT)) OR (NPWT)) OR (negative-pressure wound therapy) OR (negative pressure wound therapy)) ) AND ((Colorectal Cancer) OR (rectal) OR (colostomy)) | 229 |
| Scopus | ( TITLE ( negative-pressure ) OR TITLE ( negative AND pressure ) OR TITLE ( npwt ) AND TITLE ( colorectal ) OR TITLE ( rectal ) OR TITLE ( colostomy ) ) | 43 |
| Web of Science | (((((prophylactic negative-pressure wound therapy) OR (pinpwt)) OR (NPWT)) OR (negative-pressure wound therapy) OR (negative pressure wound therapy)) ) AND ((Colorectal Cancer) OR (rectal) OR (colostomy)) | 150 |
| Cochrane CENTRAL | (((((prophylactic negative-pressure wound therapy) OR (pinpwt)) OR (NPWT)) OR (negative-pressure wound therapy) OR (negative pressure wound therapy)) ) AND ((Colorectal Cancer) OR (rectal) OR (colostomy)) in Title Abstract Keyword | 27 |
